# Molecular confirmation of Lassa fever imported into Ghana

**DOI:** 10.4102/ajlm.v5i1.288

**Published:** 2016-04-25

**Authors:** Joseph H.K. Bonney, Edward O. Nyarko, Sally-Ann Ohene, Joseph Amankwa, Ralph K. Ametepi, Shirley C. Nimo-Paintsil, Badu Sarkodie, Prince Agbenohevi, Michael Adjabeng, Nicholas N.A. Kyei, Samuel Bel-Nono, William K. Ampofo

**Affiliations:** 1 Noguchi Memorial Institute for Medical Research, University of Ghana, Legon, Ghana; 2 37 Military Hospital, Public Health Division, Accra, Ghana; 3 World Health Organization Ghana Country Office, Accra, Ghana; 4 Disease Surveillance Department, Ghana Health Service, Accra, Ghana

## Abstract

**Background:**

Recent reports have shown an expansion of Lassa virus from the area where it was first isolated in Nigeria to other areas of West Africa. Two Ghanaian soldiers on a United Nations peacekeeping mission in Liberia were taken ill with viral haemorrhagic fever syndrome following the death of a sick colleague and were referred to a military hospital in Accra, Ghana, in May 2013. Blood samples from the soldiers and five asymptomatic close contacts were subjected to laboratory investigations.

**Objective:**

We report the results of these investigations to highlight the importance of molecular diagnostic applications and the need for heightened awareness about Lassa fever in West Africa.

**Methods:**

We used molecular assays on sera from the two patients to identify the causative organism. Upon detection of positive signals for Lassa virus ribonucleic material by two different polymerase chain reaction assays, sequencing and phylogenetic analyses were performed.

**Results:**

The presence of Lassa virus in the soldiers’ blood samples was shown by L-gene segment homology to be the Macenta and las803792 strains previously isolated in Liberia, with close relationships then confirmed by phylogenetic tree construction. The five asymptomatic close contacts were negative for Lassa virus.

**Conclusions:**

The Lassa virus strains identified in the two Ghanaian soldiers had molecular epidemiological links to strains from Liberia. Lassa virus was probably responsible for the outbreak of viral haemorrhagic fever in the military camp. These data confirm Lassa fever endemicity in West Africa.

## Introduction

Lassa virus is a single-stranded RNA virus and a member of the *Arenaviridae* family and the genus *Arenavirus*. The virus is the aetiologic agent of Lassa fever, which is an acute and often fatal illness, endemic in portions of West Africa.^1^ There are an estimated 300 000 to 500 000 cases of Lassa fever each year,^2^ with a reported mortality rate of 15% – 20% for hospitalised patients. Rodents of the genus *Mastomys* are the reservoir for the virus, which is transmitted through direct contact with materials contaminated with urine and/or droppings of infected rodents. Human-to-human transmission of the virus may give rise to nosocomial or community-based outbreaks. Subsequent to its first isolation in Lassa, Nigeria and its restricted endemicity to two geographical regions in West Africa, recent reports have shown expanded areas of spread.^3,4^ The upturn in global travel activities and international commitments in conflict and disaster situations have made the import of Lassa fever and other viral haemorrhagic fevers (VHFs) into non-endemic countries a more likely event than in the past, which has been well documented.^5,6,7,8^ Presently, an epidemic of another VHF, Ebola virus disease, has spread throughout Guinea and beyond its borders. As of December 6, 2014, a total of 17 895 Ebola virus cases, including 6394 deaths, had been reported in six West African countries (Guinea, Liberia, Sierra Leone, Nigeria, Senegal and Mali) and the Democratic Republic of the Congo.^9^

The use of molecular diagnostic tools has resulted in highly sensitive and specific tests for infectious organisms and genetic diseases. These tools, which include polymerase chain reaction (PCR) and molecular sequencing, have come in handy for the rapid identification of Lassa virus and other VHF agents from suspected cases.

In May 2013, an outbreak of acute febrile illness affecting three military personnel occurred in the Ghanaian United Nations Mission in Liberia military contingent in Zorzor, Liberia. Of the three cases, two were evacuated to a military hospital in Ghana, where molecular diagnostic methods were used to determine the causative agent. Two of the three cases were fatal.

## Research method and design

### Ethical considerations

Verbal consent was sought from the close relatives of the patients. The officers were also spoken to personally by the medical team at the 37 Military Hospital.

### Setting and patients

The index case was a male soldier with severe symptoms of malaria who died on May 11, after admission to a Level II hospital for about a week. Two male colleagues, aged 27 years (Patient 1) and 33 years (Patient 2), who were living in the same camp, subsequently developed severe fever and myalgia on May 15 (Patient 1) and 21 (Patient 2). Their medical conditions deteriorated, necessitating their medical evacuation to the 37 Military Hospital, a Level IV facility, in Accra, Ghana, on May 26, 2013 after five days of illness for Patient 1 and 11 days for Patient 2. Patient 1 died two days after admission. At the military hospital in Accra, the medical staff ruled out malaria through microscopic examination of thick and thin blood smears and focused on the clinical manifestations, which were classified as VHF. A presumptive clinical diagnosis of Lassa fever was made on the strength of previous outbreaks of Lassa fever in Liberia.^10^ To investigate these clinical suspicions, blood samples from the patients and five asymptomatic close contacts were analysed.

### Laboratory investigations

Five millilitres of blood were collected from each of the two patients (Patient 1 sample number: 60FSD_28052013; Patient 2 sample number: 59_PN_28052013) and five asymptomatic contacts (medical staff who had initially attended the two patients without barrier nursing). The samples were transported to the Virology Department of the Noguchi Memorial Institute for Medical Research at the University of Ghana in Legon, Ghana. The blood samples were processed into serum by low-speed centrifugation at 4 °C and the resultant 3 mL aliquots of sera were cryopreserved at -80 °C.

### Real-time reverse transcription-PCR Pan *Filoviridae* assay for Marburg and Ebola

Viral RNA was extracted from 140 µL of the blood samples using the QIAamp viral RNA mini kit (Qiagen, Hilden, Germany) according to the manufacturer’s instructions. A diagnostic assay developed by Panning et al.,^11^ for filovirus species and carried out with a OneStep RT-PCR reaction kit (Qiagen, Hilden, Germany) was used in a 25 µL total reaction volume, including 3 µL of the extracted RNA. The real-time diagnostic assay used five optimised large (L)-gene primers and three probes, as well as an internal control with a separate detection probe. Reactions were supplemented with 40 ng/mL bovine serum albumin and 400 mmol/L each dNTP. The primers and probes used were designed and published by Panning et al.^11^ (Table 1). All probes were synthesized by Tib-Molbiol (Berlin, Germany). Amplification in a 96-well ABI 7300 Real Time PCR System (Life Technology Holdings, Singapore) involved the following steps: 50 °C for 30 minutes; 95 °C for 15 minutes; and 45 cycles of denaturation at 95 °C for 15 seconds, annealing at 52 °C for 25 seconds and extension at 72 °C for 20 seconds. Fluorescence was measured at the end of each 52 °C annealing step.

**TABLE 1:** Reverse transcription-PCR test assays used in the laboratory investigations, Ghana, 2013.

Virus	Reagents; Primers and probes (sequence 5′ – 3′)	Target region	Reference
Filo†	OneStep RT-PCR kit (Qiagen); FiloA2.4, AAG CAT TTC CTA GCA ATA TGA TG-GT (200 nmol/L); FiloA2.2, AAG CCT TTC CTA GCA ACA TGA TGG T (200 nmol/L); FiloA2.3, AAG CAT TCC CTA GCA ACA TGA TGG T (200 nmol/L); FiloB, ATG TGG TGG GTT ATA ATA ATC ACT GAC ATG (300 nmol/L); FiloB-RAVN, GTG AGG AGG GCT ATA AAA GTC ACT GAC ATG (300 nmol/L); EBO Sud, FAM-CCG AAA TCA TCA CTI GTI TGG TGC CA-Black Hole Quencher 1 (BHQ1) (66.7 nmol/L); EBOg, FAM-CCA AAA TCA TCA CTI GTG TGG TGC-BHQ1 (66.7 nmol/L); Fam MBG, FAM-CCT ATG CTT GCT GAA TTG TGG TGC CA-BHQ1 (66.7 nmol/L)	L-gene	11
Lassa	OneStep RT-PCR kit (Qiagen); 36E2, ACC GGG GAT CCT AGG CAT TT (10 μM); LVS-339-rev, GTT CTT TGT GCA GGA MAG GGG CAT KGT CAT (10 μM)	S/GPC gene	13
Lassa	AgPath-ID One-Step RT-PCR kit (Ambion); LVL 3359D_Y+, AGA ATC AGT GAA AGG GAA AGC AAY TC (10 μM); LVL 3359G_Y+, AGA ATT AGT GAA AGG GAG AGT AAY TC (10 μM); LVL 3754A_R-, CAC ATC ATT GGT CCC CAT TTA CTA TGR TC (10 μM); LVL 3754D_R-, CAC ATC ATT GGT CCC CAT TTA CTG TGR TC (10 μM)	L-gene	18
Lassa	Power SYBR® Green RNA-to-CT™ 1-Step Kit (Life Technologies); 36E2, ACC GGG GAT CCT AGG CAT TT (10 μM); LVS-339-rev, GTT CTT TGT GCA GGA MAG GGG CAT KGT CAT (10 μM)	S/GPC gene	13

*Source*: Various

† Filoviruses including Ebola and Marburg virus.

### Gel-based Pan *Flaviviridae* assay for Yellow fever and Dengue fever

An endpoint reverse transcription (RT)-PCR protocol for the detection and identification of flaviviruses, developed by Pierre, Drouet and Deubel in 1994^12^ with a set of universal oligonucleotide primers, was used for laboratory investigation of flaviviruses present in the serum samples. These primers correspond to the 3’ non-coding region of the NS5 gene, which is highly conserved amongst the mosquito-borne flaviviruses. The OneStep RT-PCR kit (Qiagen, Hilden, Germany) was used with the following cycling conditions: reverse transcription at 50 °C for 30 minutes; initial activation step at 95 °C for 15 minutes; 45 cycles in a 3-step cycling of 95 °C for 30 seconds, 52 °C for 30 seconds and 72 °C for 30 seconds, with a cooling step of 30 seconds at 30 °C.

### Gel-based Lassa fever S-gene segment assay

A gel-based conventional RT-PCR assay was performed using ABI 2720 Thermal Cycler (Life Technology Holdings, Singapore) for the detection of Lassa virus with primers specific for regions of the S RNA segment. The RT-PCR (45 cycles) contained OneStep RT-PCR kit reagents (Qiagen, Hilden, Germany) with the sense primer 36E2 and the antisense primer LVS-339-rev, as described by Ölschläger et al.^13^ (Table 1). Cycling conditions for the RT-PCR involved the following steps: 50 °C for 30 minutes; 95 °C for 15 minutes; and 45 cycles of 95 °C for 15 seconds, 52 °C for 30 seconds and 72 °C for 30 seconds. The amplification products (expected size: 320 bp) were electrophoresed on a 2% agarose gel (peqlab Biotechnologie, Erlangen, Germany), stained with ethidium bromide and viewed under a Kodak Gel Logic 100 Imaging System (Cole-Parmer Int., Chicago, Illinois, United States).

### Lassa virus L-gene segment amplification by RT-PCR

An RT-PCR assay specific to the L-gene segment of the Lassa arenavirus on the RNA extracted from the processed blood samples. The 45 cycle RT-PCR used a AgPath-ID One-Step RT-PCR kit (Ambion, Life Technologies, Thermo Fisher Scientific, New York, New York, United States) with the primers as described by Vieth et al.^14^ (Table 1). The reaction solution consisted of: 2.5 μL nuclease-free H_2_O, 12 μL 2X RT-PCR buffer, 2 μL of the upstream and downstream primers (10 μM each), 1.5 μL 2X RT-PCR buffer enzyme mix and 5 µL DNA. Nucleic acid amplification started with a 30 minute RT step at 50 °C, then an initial denaturation step of 2 minutes at 95 °C and a 45-cycle amplification of 20 seconds at 98 °C, 30 seconds at 55 °C and 60 seconds at 72 °C. A gel was used to separate the products of the nucleic acid amplification. Images of the DNA bands were captured using a Gel Logic 100 imaging system (Eastman Kodak Company, Rochester, New York, United States). The DNA bands of nearly 400 bp in size were analysed and purified for sequencing.

### Real-time RT-PCR assay for Lassa fever

A real-time RT-PCR assay was performed using the ABI 7300/7500 Real Time PCR System (Life Technology Holdings, Singapore) for the detection of Lassa virus with primers specific for regions of the small (S) RNA gene segment. The reagent used in the preparation of the master mix for the 45-cycle RT-PCR was the Power SYBR® Green RNA-to-CT™ 1-Step Kit (Life Technologies, Carlsbad, California, United States) with the sense primer 36E2 and the antisense primer LVS-339-rev, as described in detail by Ölschläger et al.^13^ (Table 1). The amplification protocol was as follows: 48 °C for 30 minutes; 95 °C for 10 minutes; and 45 cycles of 95 °C for 15 seconds, 60 °C for 1 minute. At the end of each cycle, fluorescence was measured at 60 °C.

### Sequencing and phylogenetic analysis

The PCR products generated in the conventional RT-PCRs from the L-gene segment fragments were purified in accordance with the manufacturer’s instructions using a commercial kit (Big Dye XTerminator Purification Kit, Applied Biosystems, Carlsbad, California, United States). The purified products were sequenced on both strands using the L-gene PCR primers and an ABI Prism 3130 genetic analyser (Hitachi High Technology, Singapore). The sequences were assembled with Lasergene software (DNASTAR, Thermo Fisher Scientific, New York, New York, United States) and automated base calling was proofread by visual inspection of the electropherograms. Other published Lassa virus sequences were obtained from the National Center for Biotechnology Information’s^15,16^ GenBank (Table 2) to align with the generated sequences for phylogenetic analysis. With the use of the National Library of Medicine’s Basic Local Alignment Search Tool^17^ (BLAST) program, we compared the patients’ Lassa virus sequences with other reference Lassa virus sequences to locate regions that were similar. All sequences obtained were copied into BioEdit software (North Carolina State University, Raleigh, North Carolina, United States) and put together into an ideal sequence alignment file with the use of a multiple sequence alignment tool, ClustalW^18^ (version 2; EMBL-EBI, Cambridge, United Kingdom). We then used Molecular Evolutionary Genetics Analysis (MEGA) software (version 5.05) in a Kimura two-parameter model^19^ to infer a phylogenetic tree from the aligned nucleotide sequences following a neighbour-joining method. To establish reliability and to infer the strength of similarity between the patient’s sequence and the referenced sequences from the phylogenetic tree, a bootstrap analysis of 1000 replicates was used.

**TABLE 2:** Lassa fever viral strains used for homology and phylogenetic analysis of Ghana case strains, 2013.

Strain	Year submitted to GenBank	Location	Accession number
Las803792	2004	Liberia	AY693638
SL06-2057	2010	Sierra Leone	GU979507
Josiah	2011	Sierra Leone	JN650518
Recom_Josiah	2010	Sierra Leone	HQ688675
SL620	2003	Nigeria	AY363905
BA366	2010	Guinea	GU979513
CSF	2002	Nigeria	AY179174
Nig04-010	2004	Nigeria	AY693637
AN21366	2003	Germany	AY363904
Nig08-A47	2010	Nigeria	GU481079
SL15	2003	Germany	AY363906
Soromba R	2010	Mali	GU573542
AV	2004	Cote d’Ivoire	AF246121
Nig11-205	2011	Nigeria	JN651400
BA377	2010	Guinea	GU830840
PB1	2005	Nigeria	AJ969405
Macenta	2004	Guinea	AY628200
LP	1996	Lassa, Nigeria	U80004

## Results

### Detection and characterisation of nucleic acid in clinical specimens

**FIGURE 1:**
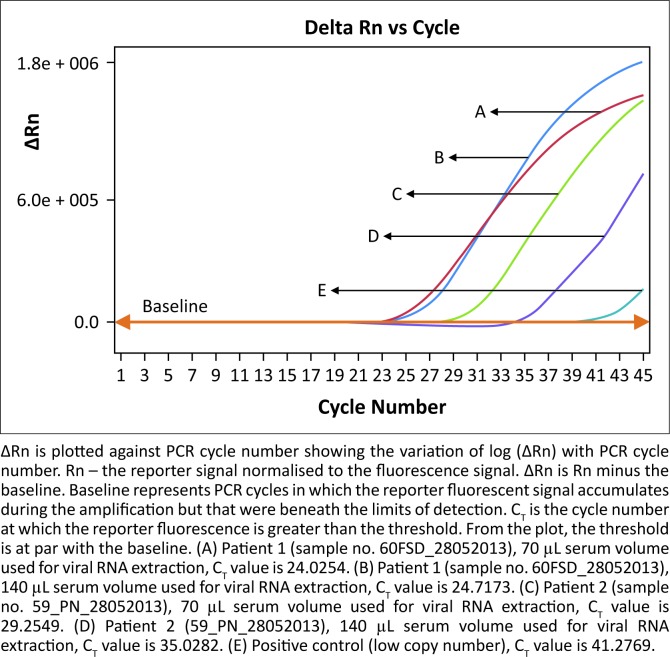
Real-time PCR amplification of patient sera and controls.

The gel-based RT-PCR flavivirus tests for Yellow fever and Dengue fever (types 1–4), as well as the real-time RT-PCR filovirus test for Marburg and Ebola, were negative (Table 3). However, the RT-PCR amplification of the S-gene segment of arenaviruses detected a 320 bp DNA band in the well with the patients’ clinical specimens. The length and position of these bands were on a par with the positive Lassa virus control (an inactivated culture supernatant of cells infected with Lassa virus strain CSF); no band was observed in the negative control (PCR-grade water) well. The real-time RT-PCR amplification of the S-gene segment of the arenavirus test produced a clear peak with a sigmoid-shaped curve for the patient’s samples, whereas no peak was observed for the negative control, which in the figure is covered by the threshold line, or baseline (Figure 1). This two-result signal indicated the presence of Lassa virus in the patients’ sera. The samples from the five asymptomatic close contacts were tested in the same assay run and no indication of the presence of Lassa virus was observed (data not shown).

**TABLE 3:** Summary of suspected patient information and their test results, Ghana, 2013.

Parameter	Patients	
Sample number	59_PN_28052013	60FSD_28052013
Gender	Male	Male
Age	33	27
Date of sample collection	May 26, 2013	May 26, 2013
Gel-based Pan *Filoviridae* (Marburg and Ebola)	Negative	Negative
Gel-based Pan *Flaviviridae* (Yellow fever and Dengue)	Negative	Negative
Gel-based *Arenaviridae* (L-gene segment)	Positive	Positive
Gel-based Lassa fever (S-gene segment)	Positive	Positive
Real-time Lassa fever	Positive	Positive

### Lassa virus L-gene segment sequencing and homology analysis

**FIGURE 2:**
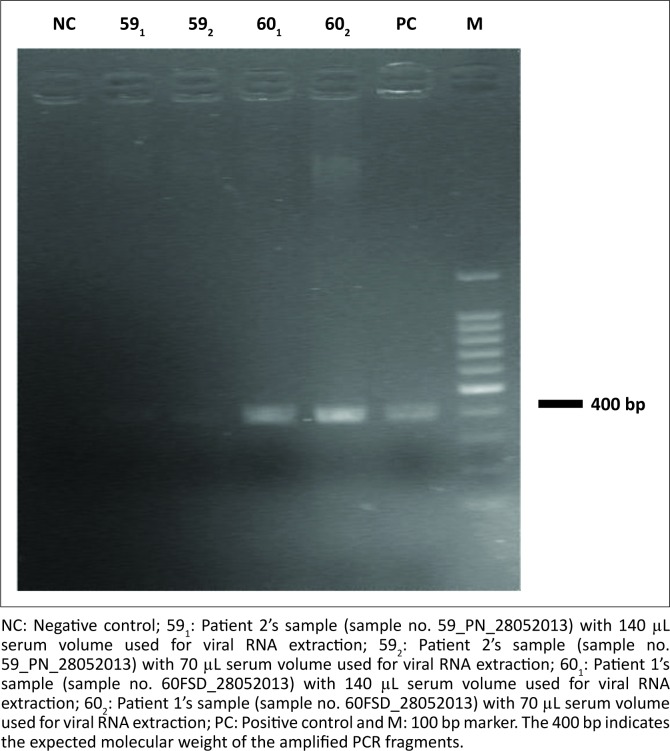
Ethidium bromide-stained 2% agarose gel image of the PCR products generated from the two patients samples run in duplicate.

The gel-based RT-PCR amplification assay for the large L-gene segment of the arenavirus showed ~400 bp products that matched the expected size (Figure 2). The comparison of the nucleotide sequences from the amplified products with the GenBank database showed that the patients’ sequences had high similarities to known Lassa virus strains. Genetically, the sequences were close to strains that had originated in Guinea and been reported in Liberia. The highest similarity (85% maximum identity in nucleotides) was to Lassa virus strain Macenta^20^ and Lassa virus strain las803792.^14^

### Phylogenetic tree analysis

**FIGURE 3:**
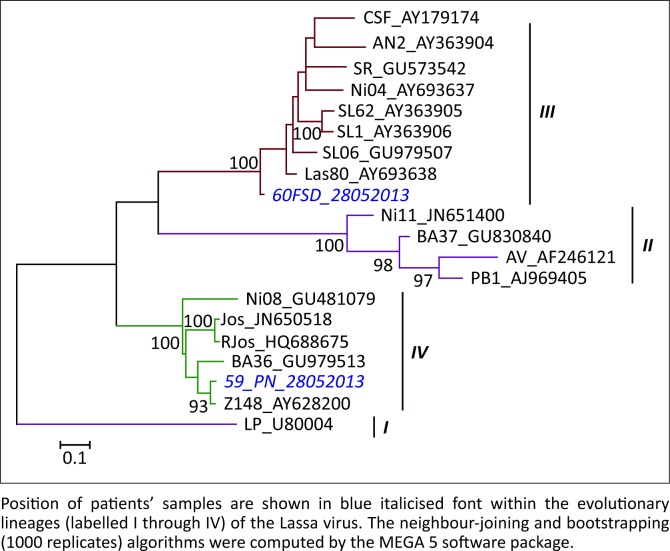
Phylogenetic relationship between the nucleotide sequences of reported Lassa virus strains and that of the two patients.

The phylogenetic analysis with GenBank data revealed that the nucleotide sequences from the patients had close phylogenetic relationships with the reported Lassa virus strains Macenta^20^ (GenBank accession number: AY628200) and las803792^14^ (GenBank accession number: AY693638) (Figure 3). The sequence data from the patient who died (Patient 1, sample no. 60FSD_28052013) was sent to GenBank and assigned accession number KF425246.

## Discussion

We identified the aetiological agent responsible for suspected cases of VHF imported into Ghana. The patients were soldiers with the Ghanaian United Nations Mission in Liberia military contingent in Zorzor, Liberia, who had had close contact earlier with another soldier suspected of dying from VHF. These patients were medically evacuated, with deteriorating health conditions, to a Level IV military hospital in Ghana, where viral nucleic acid was detected and characterised from the patients’ blood specimens. The characteristic arenavirus signal indicated on both the gel-based and real time RT-PCRs was confirmed as Lassa virus by capillary DNA sequencing.

These results confirmed Lassa virus as the aetiological agent that caused the outbreak in Liberia, where Lassa fever is known to be endemic.^21^ It has been documented that Lassa fever seems to have two geographically-separate endemic areas: the Mano River region in the west (Guinea, Sierra Leone and Liberia) and Nigeria in the east.^22^ Moreover, literature indicates that, since the initial discovery of Lassa fever in Nigeria in 1969, nosocomial outbreaks have occurred repeatedly in three localities, specifically Zorzor, Phebe and Ganta in Liberia.^23^

Molecular analyses of patients’ samples from the outbreak we report are indicative of clues regarding the source of the aetiological agent. The nucleotide sequences from the samples aligned closely with the Lassa fever strain isolated in Guinea in 2004^24^ and also reported subsequently in Liberia.^21^ The 85% proportion of alignment between GenBank-reported strains and sequences from the patients’ sera suggests a link between the source of the patients’ Lassa virus infection and the district of Zorzor in Liberia from which they were evacuated. In addition, this finding supports the assertion that persons participating in humanitarian missions or peacekeeping activities in the regions comprising Sierra Leone and Liberia are at risk for Lassa fever.^23,24,25^

Phylogenetic analysis of our patients’ sequences with GenBank-reported Lassa virus strains yielded a single parsimonious tree rooted with the prototype LP strain of Lassa virus. Our results indicate that our patients’ samples had close genetic relationships to the Macenta^20^ and las803792^14^ strains of Lassa virus, both of which were reported in Liberia but isolated in Guinea in 2004. The sequence data from the patient who died (Patient 1; sample no. 60FSD_28052013) had close homology with reported Lassa strain las803792, which was isolated from a fatal case in 2004.^14^ This observation is consistent with a study that suggested that Lassa virus strains differ in virulence potential.^1^

### Limitations

Our report was limited by our inability to conduct a battery of tests, including serological assays, for either an ideal suspected case(s) of VHF or for the five asymptomatic close contacts of the evacuated patients. That notwithstanding, this report underscores the importance of preventive measures for all visitors and workers including peacekeeping forces to endemic regions in West Africa. This is because there is currently no effective Lassa fever vaccine is available.

### Recommendations

It is recommended that medical support plans for peacekeeping operations should be built purposefully and in consideration of existing endemicity and history of endemicity in the host nations. Such support plans should be duly informed by frequently-updated research on endemic agents and other health concerns in the visiting country. Sensitisation of officers and other travellers to infectious agents in the host country and the importance of speedy reporting to health facilities when unwell should be given sufficient emphasis. This report of laboratory investigations of imported cases of Lassa fever and other documented medical fatalities on past peacekeeping operations supports the need for essential medical organisational changes in future operations. This would involve a good balance of proximity to medical care and transportation time for medical emergencies.

### Conclusion

In conclusion, it is envisaged that the importation of VHFs into non-endemic countries will increase in likelihood as a result of increased travel and international commitments in conflict and disaster situations to VHF-endemic countries in West Africa. Thus, healthcare providers should: (1) have a high index (low threshold) of suspicion for VHF amongst travellers returning from endemic areas; (2) promptly implement appropriate infection prevention and control measures; and (3) rapidly report suspected cases to avert undue nosocomial transmission.

## References

[1] Günther S, Lenz O Lassa virus. Crit Rev Clin Lab Sci. 2004; 41(4):339–390. http://dx.doi.org/10.1080/10408360490497456 15487592 10.1080/10408360490497456

[2] McCormick JB Epidemiology and control of Lassa fever. Curr Top Microbiol Immunol. 1987;134:69–78. http://dx.doi.org/10.1007/978-3-642-71726-0_3 3581899 10.1007/978-3-642-71726-0_3

[3] Sogoba N, Feldmann H, Safronetz D Lassa fever in West Africa: evidence for an expanded region of endemicity. Zoonoses Public Health. 2012;59(Suppl 2):43–47. http://dx.doi.org/10.1111/j.1863-2378.2012.01469.x 22958249 10.1111/j.1863-2378.2012.01469.x

[4] Dzotsi EK, Ohene SA, Asiedu-Bekoe F, et al The first cases of Lassa fever infection in Ghana. Ghana Med J. 2012;46(3):166–170. 23661832 PMC3645162

[5] Unit for Surveillance and Communication, Unit for Preparedness and Response, Editorial team Case of Lassa fever imported into Germany from Sierra Leone. Euro Surveill. 24 July 2006;11(30):pii=3008. 16966767

[6] Günther S, Emmerich P, Laue T, et al Imported Lassa fever in Germany: molecular characterization of a new Lassa virus strain. Emerg Infect Dis. 2000;6(5):466–476. http://dx.doi.org/10.3201/eid0605.000504 10998376 10.3201/eid0605.000504 PMC2627947

[7] Günther S, Weisner B, Roth A, et al Lassa fever encephalopathy: Lassa virus in cerebrospinal fluid but not in serum. J Infect Dis. 2001;184(3):345–349. http://dx.doi.org/10.1086/322033 11443561 10.1086/322033

[8] Schmitz H, Köhler B, Laue T, et al Monitoring of clinical and laboratory data in two cases of imported Lassa fever. Microbes Infect. 2002;4(1):43–50. http://dx.doi.org/10.1016/S1286-4579(01)01508-8 11825774 10.1016/s1286-4579(01)01508-8

[9] World Health Organization Ebola virus disease, West Africa – update [page on the Internet]. c2014 [cited 2014 Dec 06]. Available from: http://who.int/csr/don/2014_05_15_ebola/en/

[10] Frame JD, Jahrling PB, Yalley-Ogunro JE, et al Endemic Lassa fever in Liberia. II. Serological and virological findings in hospital patients. Trans R Soc Trop Med Hyg. 1984;78(5):656–660. http://dx.doi.org/10.1016/0035-9203(84)90232-3 6390808 10.1016/0035-9203(84)90232-3

[11] Panning M, Laue T, Ölschlager S, et al Diagnostic reverse-transcription polymerase chain reaction kit for filoviruses based on the strain collections of all European biosafety level 4 laboratories. J Infect Dis. 2007;196(Suppl 2):S199–204. http://dx.doi.org/10.1086/520600 17940950 10.1086/520600 PMC7109892

[12] Pierre V, Drouet MT, Deubel V Identification of mosquito-borne flavivirus sequences using universal primers and reverse transcription/polymerase chain reaction. Res Virol. 1994;145(2):93–104. http://dx.doi.org/10.1016/S0923-2516(07)80011-2 7520190 10.1016/s0923-2516(07)80011-2

[13] Ölschläger S, Lelke M, Emmerich P, et al Improved detection of Lassa virus by reverse transcription-PCR targeting the 5’ region of S RNA. J Clin Microbiol. 2010;48(6):2009–2013. http://dx.doi.org/10.1128/JCM.02351-09 20351210 10.1128/JCM.02351-09 PMC2884523

[14] Vieth S, Drosten C, Lenz O, et al RT-PCR assay for detection of Lassa virus and related Old World arenaviruses targeting the L gene. Trans R Soc Trop Med Hyg. 2007; 101(12):1253–1264. http://dx.doi.org/10.1016/j.trstmh.2005.03.018 17905372 10.1016/j.trstmh.2005.03.018

[15] Sayers EW, Barrett T, Benson DA, et al Database resources of the National Center for Biotechnology Information. Nucleic Acids Res. 2009;40(Database issue):D13–D25. 10.1093/nar/gkr1184 PMC3245031 22140104

[16] Benson DA, Karsch-Mizrachi I, Clark K, et al GenBank. Nucleic Acids Res. 2009;40(Database issue):D48–D53. http://dx.doi.org/10.1093/nar/gkn723 10.1093/nar/gkr1202 PMC3245039 22144687

[17] Altschul SF, Gish W, Miller W, et al Basic local alignment search tool. J Mol Biol. 1990;215(3):403–410. http://dx.doi.org/10.1016/S0022-2836(05)80360-2 2231712 10.1016/S0022-2836(05)80360-2

[18] Larkin MA, Blackshields G, Brown NP, et al ClustalW and ClustalX version 2. Bioinformatics. 2007;23(21):2947–2948. http://dx.doi.org/10.1093/bioinformatics/btm404 17846036 10.1093/bioinformatics/btm404

[19] Tamura K, Peterson D, Peterson N, et al MEGA5: Molecular evolutionary genetics analysis using maximum likelihood, evolutionary distance, and maximum parsimony methods. Mol Biol Evol. 2011;28(10):2731–2739. http://dx.doi.org/10.1093/molbev/msr121 21546353 10.1093/molbev/msr121 PMC3203626

[20] Hajjaj A, Chain PSG, Do LH, et al Lassa virus strain Macenta segment L, complete sequence. Accession number: AY628200; 2004.

[21] Jahrling PB, Frame JD, Smith SB, et al Endemic Lassa fever in Liberia. III. Characterization of Lassa virus isolates. Trans R Soc Trop Med Hyg. 1985;79(3):374–379. http://dx.doi.org/10.1016/0035-9203(85)90386-4 3898483 10.1016/0035-9203(85)90386-4

[22] Fichet-Calvet E, Rogers DJ Risk maps of Lassa fever in West Africa. PLoS Negl Trop Dis. 2009;3(3):e388 http://dx.doi.org/10.1371/journal.pntd.0000388 19255625 10.1371/journal.pntd.0000388 PMC2644764

[23] Ter Meulen J, Koulemou K, Wittekindt T, et al Detection of Lassa virus antinucleoprotein immunoglobulin G (IgG) and IgM antibodies by a simple recombinant immunoblot assay for field use. J Clin Microbiol. 1998;36(11):3143–3148. 9774554 10.1128/jcm.36.11.3143-3148.1998 PMC105290

[24] Fair J, Jentes E, Inapogui A, et al Lassa virus-infected rodents in refugee camps in Guinea: a looming threat to public health in a politically unstable region. Vector Borne Zoonotic Dis. 2007;7(2):167–171. http://dx.doi.org/10.1089/vbz.2006.0581 17627434 10.1089/vbz.2006.0581

[25] Khan SH, Goba A, Chu M, et al New opportunities for field research on the pathogenesis and treatment of Lassa fever. Antiviral Res. 2008;78(1):103–115. http://dx.doi.org/10.1016/j.antiviral.2007.11.003 18241935 10.1016/j.antiviral.2007.11.003

